# Network Pharmacology Integrated Molecular Docking and Dynamics to Elucidate Saffron Compounds Targeting Human COX-2 Protein

**DOI:** 10.3390/medicina59122058

**Published:** 2023-11-22

**Authors:** Aarif Ali, Amir Bashir Wani, Bashir Ahmad Malla, Jagadeesha Poyya, Nawab John Dar, Fasil Ali, Sheikh Bilal Ahmad, Muneeb U. Rehman, Ahmed Nadeem

**Affiliations:** 1 Division of Veterinary Biochemistry, Faculty of Veterinary Sciences & Animal Husbandry, SKUAST-K, Shuhama, Alusteng, Srinagar 190006, India; 2 Genome Engineering and Societal Biotechnology Lab., Division of Plant Biotechnology, SKUAST-K, Shalimar, Srinagar 190006, India; waniamir1647@skuastkashmir.ac.in; 3 Department of Biochemistry, School of Biological Sciences, University of Kashmir, Hazratbal, Srinagar 190006, India; bashirmalla04@gmail.com; 4 SDM Research Institute for Biomedical Sciences, Dharwad 580009, India; 5 SALK Institute for Biological Studies, La Jolla, San Diego, CA 92037, USA; nawabdar@gmail.com; 6 Department of Studies and Research in Biochemistry, Mangalore University, Mangalore 571232, India; 7 Department of Clinical Pharmacy, College of Pharmacy, King Saud University, Riyadh 11451, Saudi Arabia; mrehman1@ksu.edu.sa; 8 Department of Pharmacology and Toxicology, College of Pharmacy, King Saud University, Riyadh 11451, Saudi Arabia

**Keywords:** inflammation, COX-2, saffron, docking, simulation, enrichment analysis

## Abstract

***Background and Objectives***: Cyclooxygenase-2 (COX-2) is mostly linked to inflammation and has been validated as a molecular target for treating inflammatory diseases. The present study aimed to identify novel compounds that could inhibit COX-2, which is associated with various diseases including inflammation, and in such a scenario, plant-derived biomolecules have been considered as attractive candidates. ***Materials and Methods***: In the present study, physiochemical properties and toxicity of natural compounds/drugs were determined by SWISSADME and ProTox-II. In the present study, the molecular docking binding features of saffron derivatives (crocetin, picrocrocin, quercetin, safranal, crocin, rutin, and dimethylcrocetin) against human COX-2 protein were assessed. Moreover, protein-protein interactions, topographic properties, gene enrichment analysis and molecular dynamics simulation were also determined. ***Results***: The present study revealed that picrocrocin showed the highest binding affinity of −8.1 kcal/mol when docked against the COX-2 protein. PROCHECK analysis revealed that 90.3% of the protein residues were found in the most favored region. Compartmentalized Protein–Protein Interaction identified 90 interactions with an average interaction score of 0.62, and the highest localization score of 0.99 found in secretory pathways. The Computed Atlas of Surface Topography of Proteins was used to identify binding pockets and important residues that could serve as drug targets. Use of WEBnmα revealed protein dynamics by using normal mode analysis. Ligand and Receptor Dynamics used the Molecular Generalized Born Surface Area approach to determine the binding free energy of the protein. Gene enrichment analysis revealed that ovarian steroidogenesis, was the most significant enrichment pathway. Molecular dynamic simulations were executed for the best docked (COX-2-picrocrocin) complex, and the results displayed conformational alterations with more pronounced surface residue fluctuations in COX-2 with loss of the intra-protein hydrogen bonding network. The direct interaction of picrocrocin with various crucial amino-acid residues like GLN^203^, TYR^385^, HIS^386 and 388^, ASN^382^, and TRP^387^ causes modifications in these residues, which ultimately attenuates the activity of COX-2 protein. ***Conclusions***: The present study revealed that picrocrocin was the most effective biomolecule and could be repurposed via computational approaches. However, various in vivo and in vitro observations are still needed.

## 1. Introduction

Inflammation is the biological response of the body to harmful stimuli and can be associated with pathogens, tissue damage, irritation, infection, and toxic compounds [1]. Inflammatory reactions are a non-specific defense against cell injury; their hallmarks are redness, swelling, and pain, and they are associated with the release of various inflammatory mediators including histamines, interleukins (ILs), bradykinin, leukotrienes, prostaglandins (PGs), and enzymes [2,3,4]. Under normal conditions, inflammation is restricted to affected tissues; however, in some cases, it may become uncontrollable, such as in cancers, asthma, diabetes mellitus, Alzheimer’s disease, and nephropathy [5,6,7,8]. Therapeutic interventions involve the use of non-steroidal anti-inflammatory drugs (NSAID) and narcotic analgesic agents. In the management and treatment of inflammation, cyclooxygenase 2 (COX-2) inhibitors are the main prominent molecules. The COX-2 enzyme is the main prostanoid (PG, prostacyclin, and thromboxane) synthesis enzyme, having two isoforms (COX-1 and COX-2) each with two activities like oxygenase and peroxidase activity, and it converts 20 carbon arachidonic acids into PGH_2_ through a short-lived PGG_2_ intermediate [2,3,4,9]. COX-1 is a constitutive protein, whereas COX-2 is an inducible protein; both the proteins are active in dimeric form, and their monomeric form consists of three domains: the epidermal growth factor (EGF) domain at N-terminal, the heme-peroxidase domain at the core, and the membrane anchor domain [10]. COX enzyme, primarily COX-2, becomes an important target for various anti-inflammatory drugs by causing amino acid substitution [11]. Several anti-inflammatory drugs like celecoxib, naproxen, celecoxib, ketorolac, acetylsalicylic acid, etoricoxib, and indomethacin are primarily used to treat inflammation [12,13,14]. All these drugs are also associated with various side effects and resistance development [15,16]. Therefore, we move towards naturally occurring herbal drugs as they act as precursors of various synthetic drugs to cure different diseases including inflammatory disease [17,18,19]. Plant secondary metabolites also regulate various biological activities like cancer, anti-bacterial, inflammation, anti-viral, migration, proliferation, and cell death [20,21,22,23]. Many natural products (culinary mushrooms, flavonoids, terpenoids, hyperforin, phenols, and alkaloids) have already been discovered that inhibit COX-2 expression/activity and exhibit fewer side effects [24,25,26].

In the traditional system of medicine, saffron (*Crocus sativus*) has been used as an alternative to treat various diseases in addition to its properties of flavoring, tasting, and coloring food [27]. Saffron has been widely found in Iran, Turkey, India, Italy, Greece, Egypt, Azerbaijan, Spain, and France [27,28]. Saffron is an ancient medicinal plant that possesses antioxidant, immunomodulatory, and anti-inflammatory properties [29,30,31]. Saffron has been used for the treatment of diabetes, coronary artery disorders, colorectal cancer, asthma, neurodegenerative diseases, and bronchitis [32,33,34]. The health-promoting medicinal properties of saffron are mainly due to its prominent chemical constituents such as crocins, safranal, crocetins, and carotenoids (α and β carotene, lycopene, and zeaxanthin) [35,36,37]. 

Recent developments in computational methods have been significant in developing the rationale for identifying natural compounds that can be used to target inflammatory proteins of interest. Computational approaches have the tremendous potential to repurpose plant compounds for drug design and development. Here, keeping in view the above-mentioned issues, computational methods were used to identify potential COX-2 inhibitors from compounds of saffron. In the present study, absorption, distribution, metabolism, excretion, and toxicity (ADMET) analysis of saffron phytocompounds and synthetic drugs was carried out. Moreover, we also aimed to determine the sequence alignment, molecular modeling, molecular docking, protein–protein interactions (PPIs), gene enrichment analysis, and topological properties of the protein. Further, in the present study, molecular dynamics simulations (MDs) of the topmost protein–ligand complex were performed.

## 2. Methods and Material

### 2.1. Ligand Selection

In the present study, crocetin, picrocrocin, quercetin, rutin, safranal, crocin, and dimethyl crocetin were the selected phytocompounds, whereas aspirin and rofecoxib were the synthetic drugs. The ligand’s three-dimensional (3D) structures were retrieved from PubChem (http://pubchem.ncbi.nlm.nih.gov/compound/ accessed on 7 June 2023).

### 2.2. ADMET Analysis

In the present study, the physiochemical and pharmacokinetics properties of selected natural compounds and drugs were evaluated through ADMET. SWISSADME, an online, easily available web server (http://www.swissadme.ch/ accessed on 7 June 2023), was used for determining the drug-like properties of molecules [38]. The smiles of ligands (obtained from PubChem) were then entered in input of SWISSADME to determine their ADMET characteristics. All the compounds were screened for Lipinski’s rule of five (RO5) that includes molecular weight less than 500 g/mol, LOGP < 5, topological polar surface area (TPSA) score of <140 Å^2^, and a number of hydrogen bond acceptors and donors less than 10 and 5, respectively [39]. The compounds with better values of leadlikeness, BBB (blood–brain barrier), bioavailability, gastrointestinal (GI) absorption, and synthetic accessibility were selected for further analysis.

### 2.3. ProTox-II

ProTox-II is an online web server used for determining the toxicity of selected compounds (https://ox-new.charite.de/protox_II/ last accessed on 7 June 2023). The evaluation of the toxicity of natural compounds is an essential part of drug design and development. In this study, different parameters were considered for determining toxicity, including class of toxicity, lethal dose (LD_50_), hepatotoxicity, immunotoxicity, mutagenicity, carcinogenicity, and cytotoxicity [40]. The predicted LD_50_ was obtained for crocetin (4300 mg/kg), picrocrocin (55 mg/kg), quercetin (159 mg/kg), rutin (5000 mg/kg), aspirin (250 mg/kg), and rofecoxib (2240 mg/kg). Based on the levels of toxicity, different classes were constituted, i.e., class I and II (fatal), class III (could prove toxic), class IV/V (could be harmful), and class VI (non-harmful) [40].

### 2.4. PROCHECK

The PROCHECK tool was used to determine the accuracy and quality of the selected protein model (http://saves.mbi.ucla.edu/ accessed on 7 June 2023) [41,42]. 

### 2.5. Selection of Protein

COX-2 with PDB ID 5f19 served as the target protein, the 3D crystal structure of which was obtained from the RCSB protein databank (http://www.rccsb.org/pdb/ accessed on 7 June 2023).

#### 2.5.1. Protein Preparation

AutoDock Vina (UCSF-Chimera©, version 4.2.6, Torrey, Mesa, CA, USA) was used for docking analysis, which was performed as per the protocol mentioned by [43]. The target protein was prepared in AutoDock Vina by performing various steps that included the removal of water molecules from the protein first, followed by the addition of polar-charged hydrogens, and lastly addition of Gasteiger charges. In AutoDock Vina, the protein was selected as a macromolecule and the compounds as ligands. During analysis, both ligands and the target protein were saved in the pdbqt format separately.

#### 2.5.2. Grid Dimensions

In AutoDock Vina, the center dimensions (x = 22.683, y = 40.790, and z = 39.382) and grid size of 40 × 40 × 40 with 0.375Å spacing were set. In the preparation of the configuration file during the docking process, information on both ligands and protein was used, with both candidate entities considered rigid. After completion of the docking analysis, 10 log files of the protein-ligand complex were produced with the 10 best-docked poses. For ligand–protein interaction visualization, Discovery Studio (version 2.4.1, 2016) was used.

### 2.6. Inhibition Constant

The inhibition constant (Ki) was calculated from the change in binding energy (ΔG in kcal/mol) formula:

Ki (µM) = exp (ΔG/RT)

where R = gas constant in kcal/mol/K^−1^ (1.98 × 10^−3^) and T = temperature in K (298.15).

### 2.7. Compartmentalized Protein–Protein Interaction Database (ComPPI)

A database that provides qualitative information on protein–protein interaction networks and their localizations was used (http://ComPPI.Linkgroup.hu/ accessed on 7 June 2023) [44]. ComPPI is a multi-species database that includes *S. cerevisiae*, *C. elegans*, *D. melanogaster*, and *H. sapiens*. ComPPI provides two novel scores (Interaction and Localization) that determine the probability of data correctness. The Localization Score is determined by the category of subcellular localization data (experimental, unknown, or predicted) as well as the number of resources. The Interaction Score outlines the likelihood of a protein–protein interaction’s subcellular localization and is dependent on the consensus of the interacting proteins’ compartment-specific Localization Scores.

### 2.8. Computed Atlas of Surface Topography of Proteins (CASTp)

The detailed topographic characteristics of the target protein were identified through this server (http://sts.bioe.uic.edu/ accessed on 7 June 2023). In the server input, the PDB ID or PDB format of the protein can be uploaded, and this tool is based on the alpha-shape method with a default probe radius of 1.4 Å.

### 2.9. WEBnm@

This tool provides simple automated computation analysis of low-frequency normal protein modes (http://apps.cbu.uib.no/WEBnm@/ accessed on 7 June 2023). This server enables us to understand the large amplitude movements that a protein undergoes. In computing the normal modes, this tool uses an elastic network model (ENM) with a C-alpha force field [45]. The diagonalization of the matrix of the second components of the energy parameter about the atom displacements in mass-weighted coordinates (Hessian matrix) constitutes a normal mode analysis (NMA). The Hessian matrix’s eigenvectors are the normal modes, and its eigenvalues are the squares of the related frequencies. In the present study, Hinsen’s approximate normal modes computation approach was used [45]. This approach accurately captures low-frequency domain motions at a low computing cost.

### 2.10. Ligand and Receptor Dynamics (LARMD)

Based on normal mode analysis (NMA), the LARMD tool (http://chemyang.ccnu.edu.cn/ accessed on 7 June 2023) was used to perform simulation analysis for 1 nanosecond MD time explicitly along with water. In conventional molecular dynamics, the estimation of free energy was carried out by using the LARMD server. Gibb’s free binding energy (DG bind) was calculated through conformational entropy (TDS conf) and salvation energy entropy (TDS sol) [46]. To determine entropy and enthalpy, Molecular Generalized Born Surface area (MM/GBSA) and Empirical approaches were used [47,48]. 

### 2.11. Gene Enrichment Analysis

ShinyGO is a graphical gene enrichment tool that provides valuable insights about enrichment results and gene characteristics, pathways, and protein interactions (http://bioinformatics.sdstate.edu/go/ accessed on 7 June 2023). ShinyGO operates on an amalgamation of R/Bioconductor programs and comprehensive network data along with annotation obtained from a variety of sources. This user-friendly, graphical application has made it easier to investigate gene functions and pathways in a wide range of species, including 59 plants, 256 animals, 115 archeal, and 1678 bacterial species. A Chi-squared test is used to compare the distribution of background genes on the chromosomes to the rest of the background genes in the entire genome together. The fold enrichment is calculated by dividing the proportion of genes in the list that are involved in a specific pathway by the relevant background percentage. For GO enrichment, the ShinyGO server and the Gene Ontology (GO) database were utilized at a false discovery rate (FDR) of <0.01. 

### 2.12. Molecular Dynamics Simulation

In the present study, molecular dynamics (MD) simulation on the dock complex of COX-2 with picrocrocin (best-docked protein–ligand complex) was performed via the Desmond program (Schrodinger, LLC). For each solved protein–ligand interaction, a simple point charge (SPC) method was used in this system [49]. Na^+^ and Cl^−^ ions were added to each system to neutralize the charge. For the minimization and relaxation of the system, the NPT (a number of atoms (N), pressure (P), and temperature (T) ensemble from the Desmond program were used. During the retraining of the complex with picrocrocin, the system was first equilibrated by using an NVT ensemble for 200 ns. Using an NPT ensemble, a short run for equilibration and minimization was performed for 20 ns. To ensure reliable results during the study, the chain coupling method of Nose–Hoover was used to set up the NPT ensemble and run at 27 °C and a relaxation time of 5.0 ps under a pressure of 1 bar [50]. In this experiment, a 2 fs time step was used. The Martyna–Tuckermann–Klein barostat method was used to control the pressure with 2 ps relaxation time. The final run of production was performed for a 200 ns time scale. The stability of the MD simulations was calculated by using various parameters like root mean square deviation (RMSD), root mean square fluctuation (RMSF), no. of hydrogen bonds, radius of gyration (Rg), and solvent accessible surface area (SASA).

## 3. Results

### 3.1. ADMET Properties

From the ADMET analysis, it was found that all the compounds followed Lipinski’s RO5 except rutin (molecular weight 610.52 g/mol, TPSA greater than 140 Å^2^, number of hydrogen bond acceptors >10, and donors > 5). In the plasma membrane, a TPSA score of <140 Å^2^ indicates significant permeability. In the present study, all the selected compounds had a TPSA score of less than 140 Å^2^ except rutin, which had TPSA value of 269.63 Å^2^. In this study, the selected compounds all had molar refractivity (MR) scores within the acceptable range (40–130) except rutin, which had an MR score of 141.8. In this study, all the studied compounds had lipophilicity scores (XLOGP) ranging from 5.41 to −0.50, with crocetin having a score >5. Similarly, iLOGP scores of selected compounds varied from 0.46 to 3.33 respectively. All the selected compounds except rutin showed high bioavailability scores. The gastrointestinal (GI) absorption of all the studied compounds was high except rutin, which showed lower GI absorption. The blood–brain barrier (BBB) determines the capability of compounds to cross the BBB, and in this study, none of the selected compounds showed the ability to cross the BBB. In this study, all the compounds except quercetin showed no p-glycoprotein substrate (Pgp) activity. Cytochrome P450 (CYP450) inhibitors are used to determine whether a compound is going to inhibit CYP450 and its isoforms. Skin permeability (log Kp) is an important parameter in the development of transdermal drug delivery, and scores >2.5 determine lower permeability to skin. Table 1 shows the findings of the ADMET analysis.

### 3.2. ProTox-II Analysis

**Figure 1:**
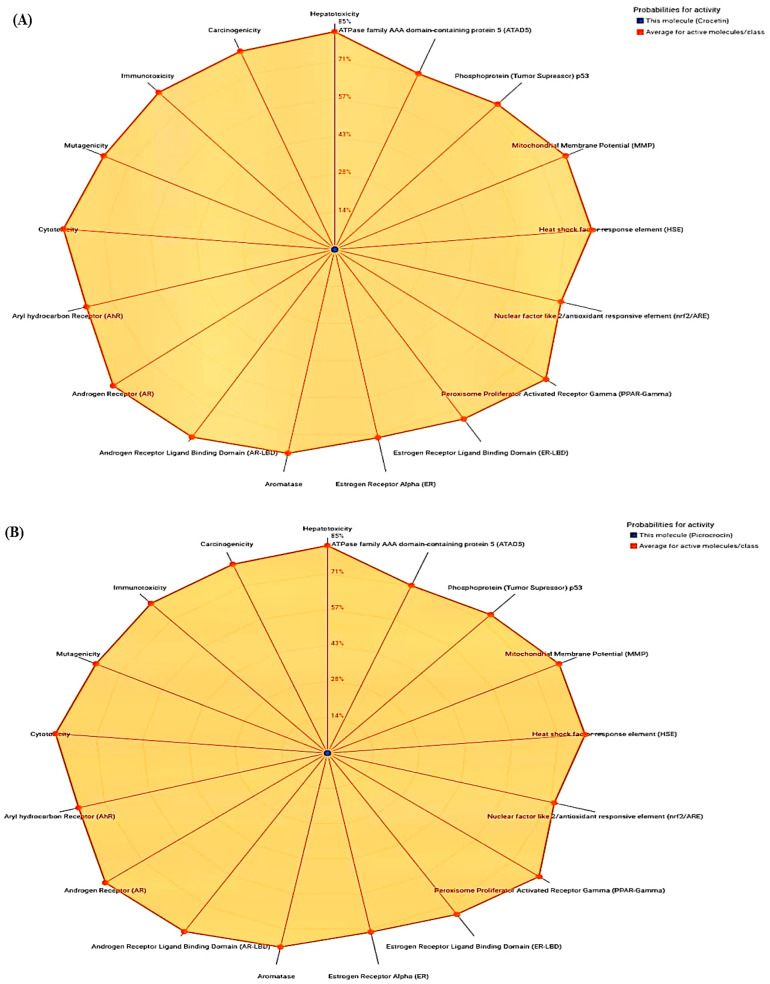

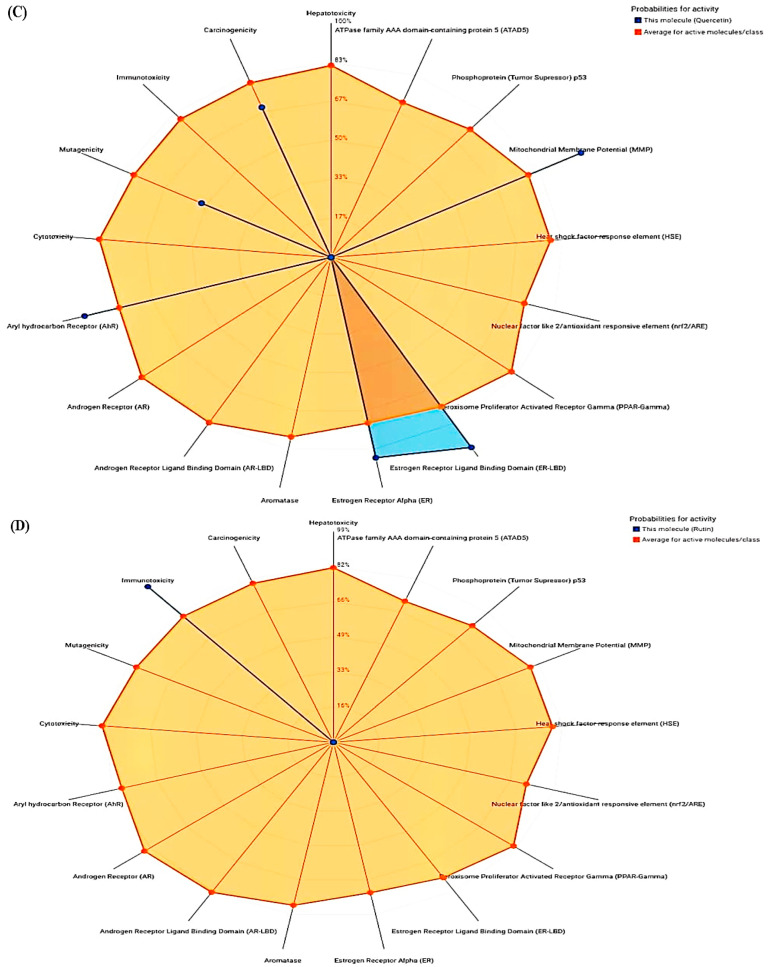

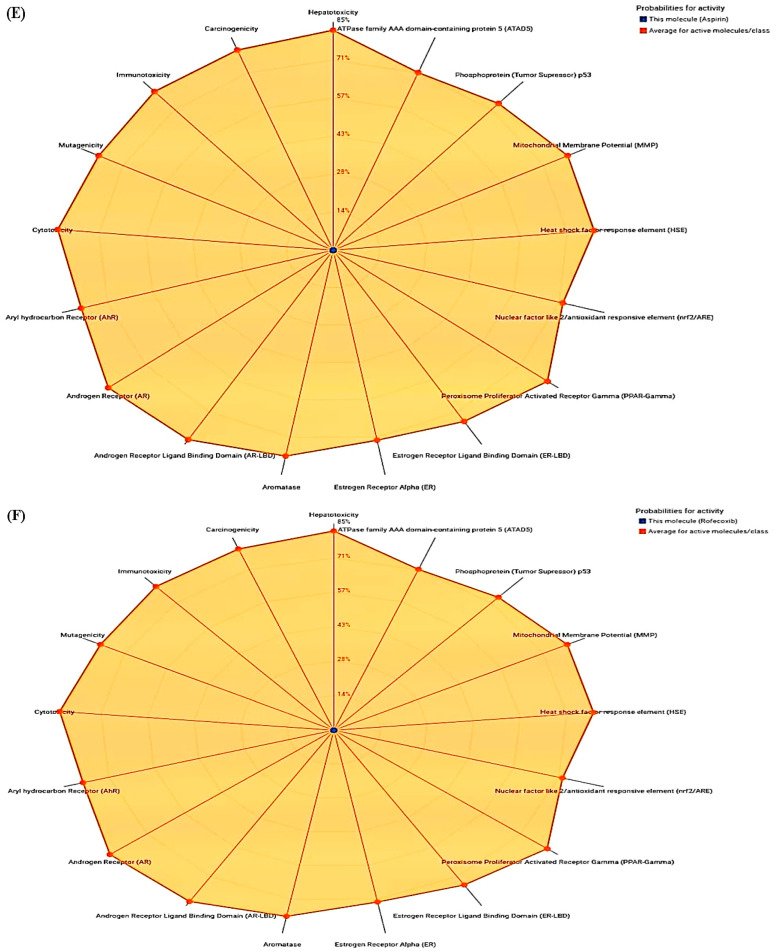
Radar toxicity chart analysis: (**A**) Crocetin, (**B**) Picrocrocin, (**C**) Quercetin, (**D**) Rutin, (**E**) Aspirin, (**F**) Rofecoxib.

The findings of the present study revealed that crocetin, aspirin, and rofecoxib were inactive for all the toxicities. However, quercetin showed active toxicity for carcinogenicity and mutagenicity and was inactive for the rest. Moreover, rutin showed inactive activity for various toxicities except for immunotoxicity. Table 2 and Figure 1A–F show the toxicity analysis scores of selected compounds.

### 3.3. Molecular Docking

In the present study, docking analysis revealed that picrocrocin had the highest binding affinity of −8.1 kcal/mol. Among the drugs, rofecoxib showed the highest binding affinity of −8.3 kcal/mol, whereas aspirin reported the lowest (6.1 kcal/mol). The inhibition constant (Ki) in µM of selected saffron compounds was determined/calculated from binding affinity. Among the compounds, picrocrocin had the highest Ki value of 13.68 µM, whereas safranal reported the lowest (11.51 µM). In the present study, Table 3 shows the binding affinity and Ki of compounds and drugs.

**Figure 2:**
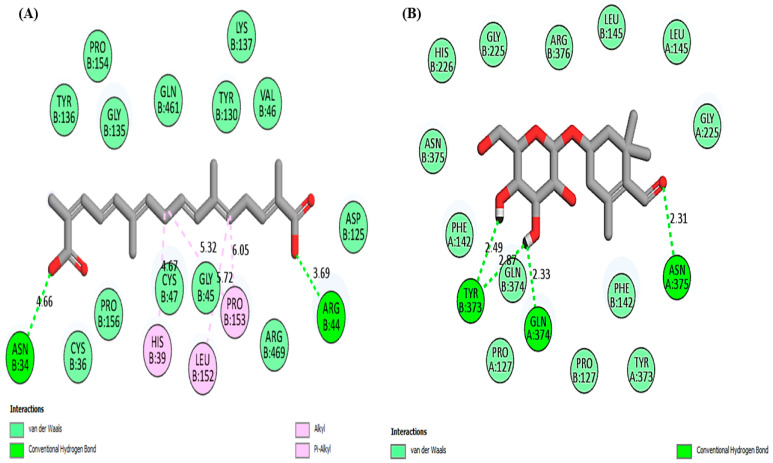

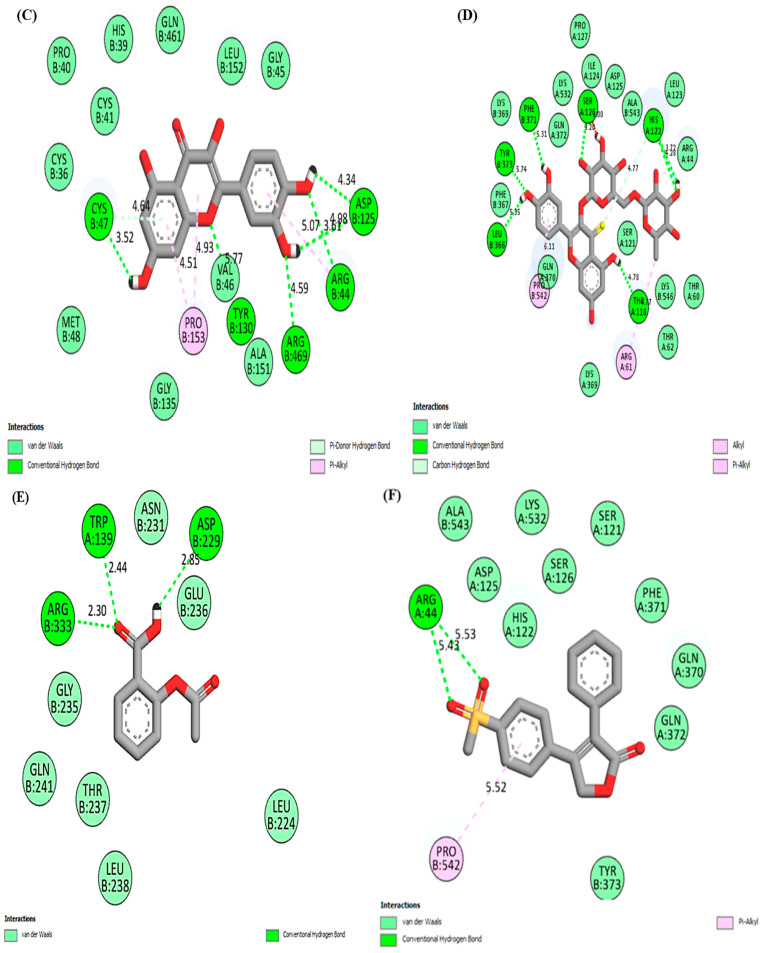
Molecular docking analysis: (**A**) Crocetin, (**B**) Picrocrocin, (**C**) Quercetin, (**D**) Rutin, (**E**) Aspirin, (**F**) Rofecoxib.

The graphical representation of 2D interactions of the COX-2 protein when docked with selected saffron compounds and known drugs are shown in Figure 2. The results showed that the compounds form different types of interactions with the amino acid residues of COX-2 protein, as shown in Table 4.

### 3.4. PROCHECK

**Figure 3:**
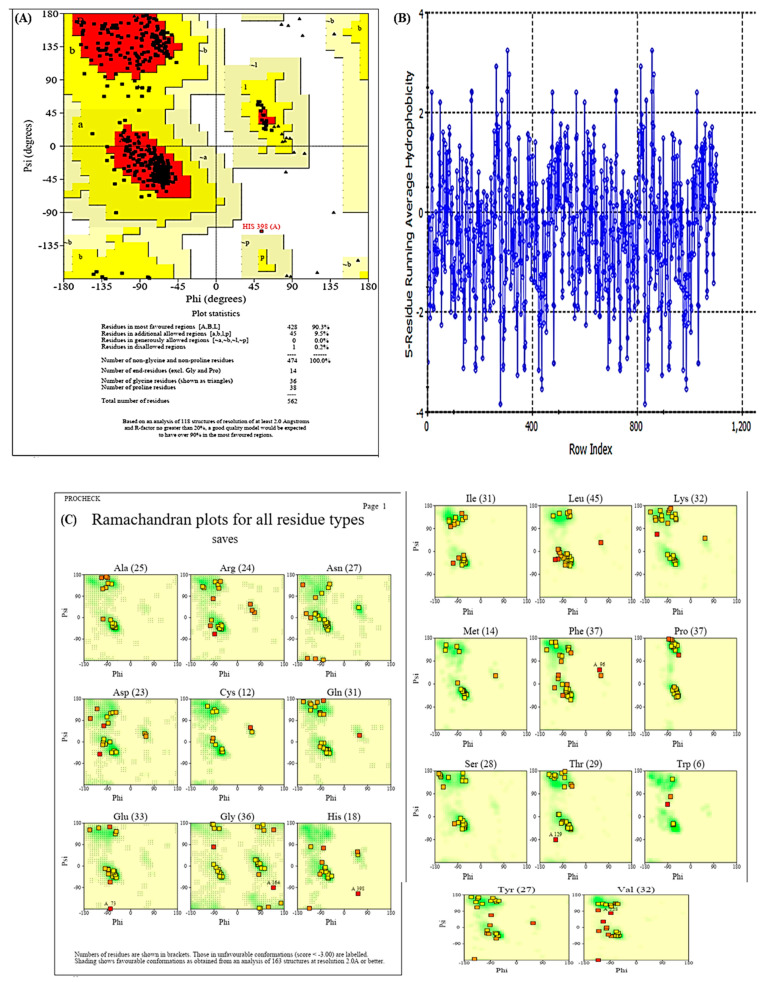
PROCHECK analysis: (**A**) Ramachandran plot statistics, (**B**) Hydrophobicity plot, (**C**) Ramachandran plot for all residues.

The PROCHECK server helps us to evaluate the 3D geometry of protein structures, and it provides results for residues with areas represented by different colors such as red (favored), yellow (additionally allowed), pale yellow (generously allowed), and white (disallowed), as shown in Figure 3A. In human COX-2, 562 residues were found, and out of those, 428 (90.3%) residues were lying in the most favored region (A, B, L). About 45 (9.5%) residues lay in the additional allowed regions (a, b, l, p), none in generously allowed regions, and 1 (0.1%) in disallowed regions (Figure 3A). In human COX-2 protein, we found 474 (100%) non-proline and non-glycine residues, while at the end, the number of residues (except glycine and proline) were only 14. The proline and glycine residues are shown as triangles and are 38 and 36, respectively (Figure 3A). In this study, Figure 3B illustrates the hydrophobicity plot of the human COX-2 protein. The Ramachandran plots for all residue types are represented in Figure 3C. The number of amino acid residues is shown in brackets and those in unfavorable conformations (score < −3.0) are labeled. Favorable conformations are illustrated by shaded regions based on analyzing a total of 163 structures at a resolution of 2.0Å or better. Shading shows favorable conformations as obtained from an analysis of 163 structures at a resolution 2.0Å or better. 

### 3.5. ComPPI

**Figure 4:**
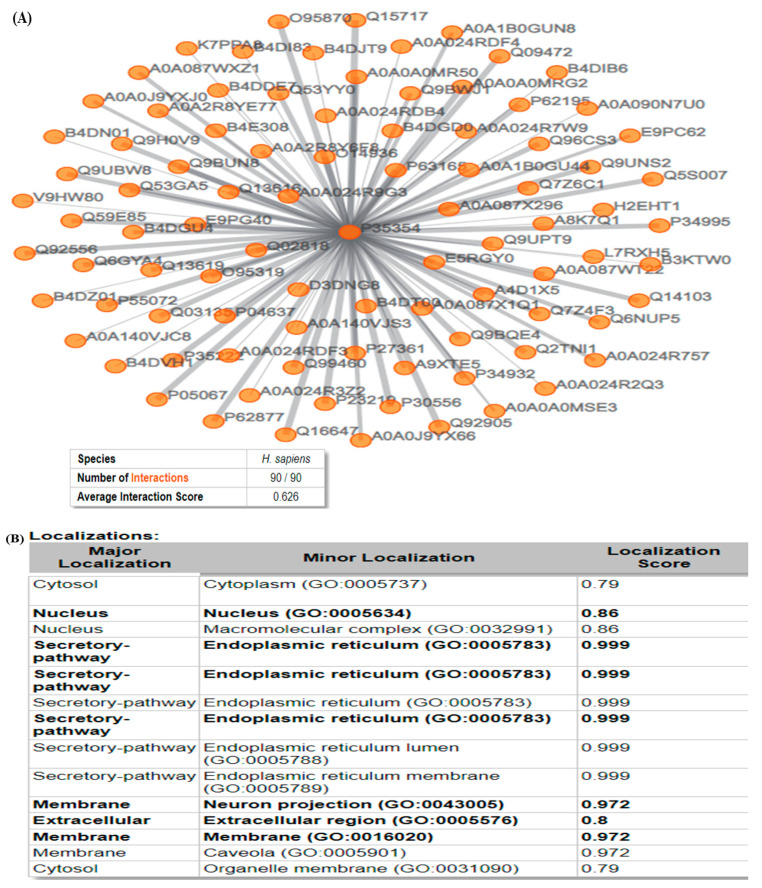
ComPPI analysis: (**A**) First-neighbor network visualization COX-2 protein, (**B**) Major and Minor localizations with scores.

In the present study, Figure 4A shows the first-neighbor network showing the interaction map, and the width of the edges is proportional to the corresponding localization score. In the present study, the number of interactions was 90 and the average interaction score was 0.626. In this study, COX-2 showed an interaction score of 1 with Amyloid-Beta A4, Caveolin-1, Prostacyclin Synthase, Derlin-1, Vip36-Like Protein, Nucleobindin-1, Prostaglandin G/H Synthase 1, Selenoprotein S, Mitogen-activated protein kinase 3, etc. (Figure 4B). The localization of the COX-2 protein is illustrated in Figure 4B, wherein the highest score was found in secretory pathways (0.99) and the lowest in cytosol (0.79).

### 3.6. CASTp

**Figure 5:**
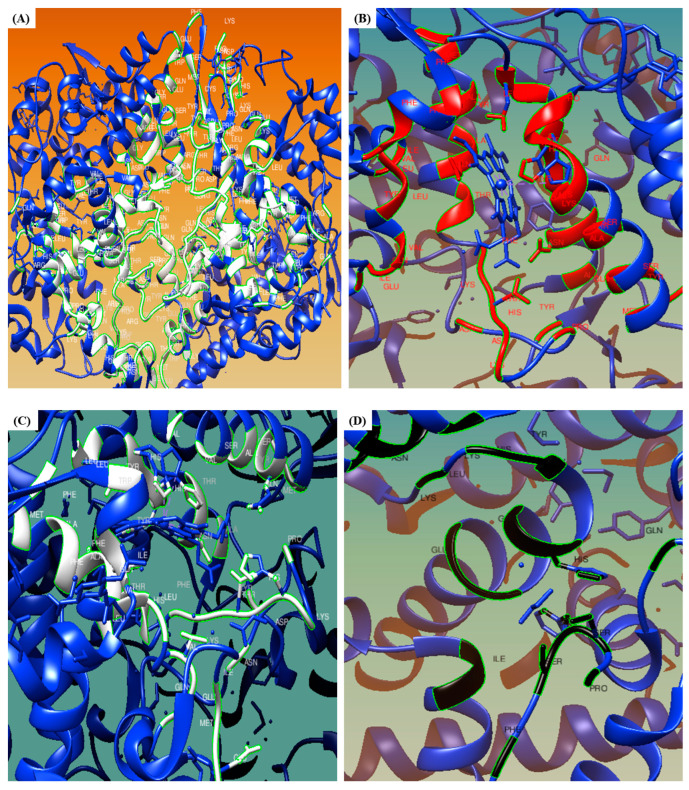

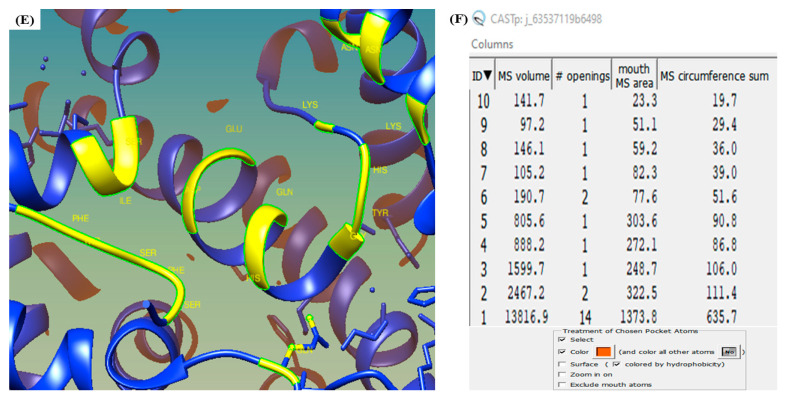
CASTp server analysis: (**A**–**E**) Different representations of pocket panel, (**F**) CASTp data.

In the present study, Figure 5 illustrates the five best binding pockets on the COX-2 protein that are automatically recognized by the CASTp tool. Figure 5A represents the top-most binding pocket of COX-2 protein, whereas Figure 5B–D indicate the second, third, fourth, and fifth binding pockets, respectively. The pocket panel shows many important residues that are located in the pocket, and these pockets could serve as targets for drugs. The imprint of the pockets is illustrated in white (Figure 5A), red (Figure 5B), white (Figure 5C), black (Figure 5D), and yellow (Figure 5E), respectively. The CASTp data showing the volume, openings, and area of the binding pockets, along with their respective positions, are illustrated in Figure 5F.

### 3.7. WEBnmα

**Figure 6:**
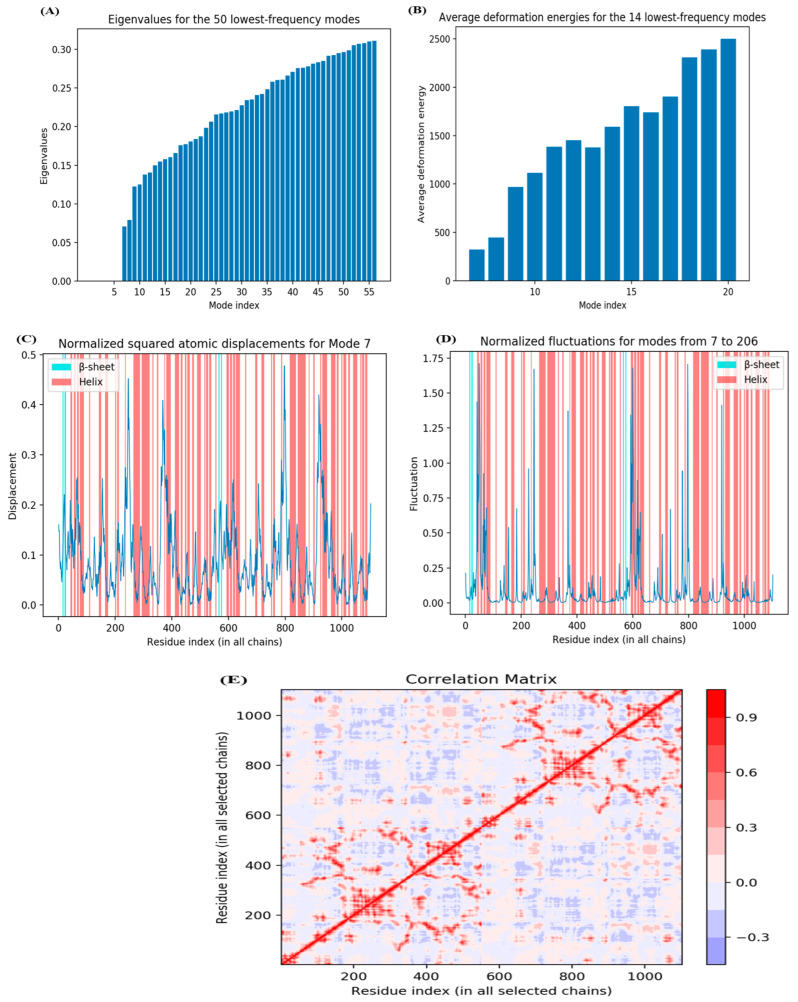
WEBnmα analysis: (**A**) Eigenvalues, (**B**) Average deformation energies, (**C**) Atomic displacement, (**D**) Fluctuation, (**E**) Covariance map.

In the present study, the eigenvalues for the 50 lowest-frequency modes starting from mode 7 are illustrated in Figure 6A. In this study, the 14 lowest-frequency modes (7–20) had average deformation energies of 323.96, 448.04, 968.32, 1113.79, 1384.79, 1451.23, 1377.34, 1589.30, 1803.78, 1737.89, 1901.58, 2306.34, 2389.80, and 2497.08, respectively (Figure 6B).

Eigenvalues and deformation energies depict the energy related to every mode and are inversely associated with the amplitude of motion expressed by the representative modes. Along all the normal modes, the protein structure moves at once. The atomic displacement of the protein molecule is shown in Figure 6C. The square of the atomic displacement of each C-alpha atom (mode 7) is normalized, and the highest values correspond to the most displaced regions. Cluster peaks represent the significantly displaced regions on the plots, whereas isolated peaks indicate local flexibility in a low-density region (N or C-terminal).

In this study, Figure 6D represents the normalized fluctuations from mode 7 to 206, respectively. The sum of atomic displacement in each of the 200 lowest frequency modes weighted by the inverse of their corresponding eigenvalues gives the fluctuation plots; thus, in the present study, we found the highest atomic fluctuation to be 1.71 and the lowest to be 0.01. The correlation matrix describes the correlated movement of the C-alphas of protein, as illustrated in Figure 6E, wherein the covariance matrix indicates the coupling between a pair of residues, i.e., red (correlated), blue (anti-correlated), and white (uncorrelated), respectively. The coupling of two residues in the proteins ranged from −1 (anti-correlated) to 1 (correlated) and 0 (uncorrelated). The color of the sticks represents indicate correlations, i.e., red (positive) and blue (negative).

### 3.8. LARMD

**Figure 7:**
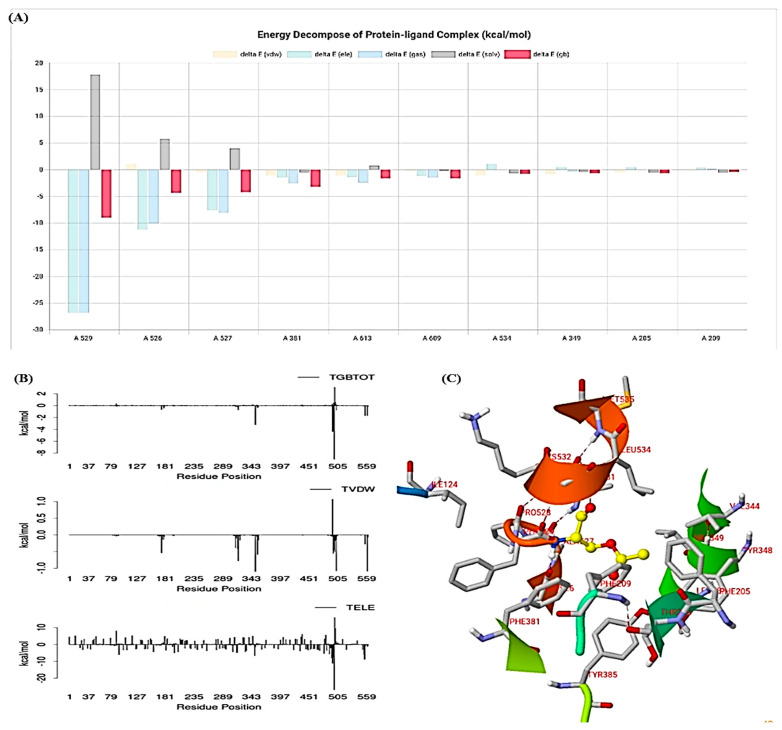
LARMD analysis: (**A**) Decomposition energy, (**B**) MM/PB(GB)SA analysis, (**C**) Binding residues.

In the present study, the LARMD server provided the results of decomposition energy (kcal/mol) of the protein molecule, as illustrated in Figure 7A. The findings of MM/PB(GB)SA, consisting of van der Waals contribution (VDW), electrostatic energy (ELE), total gas phase energy (GAS), and polar and non-polar contribution to solvation (PBSL/GBSOL) of the residues, are shown in Figure 7B. Furthermore, the final binding free energy (deltaPB/deltaGB) was determined from PBTOT/GBTOT and entropy (TS). The MM/PB(GB)SA results are shown in Table 5.

### 3.9. ShinyGO

**Figure 8:**
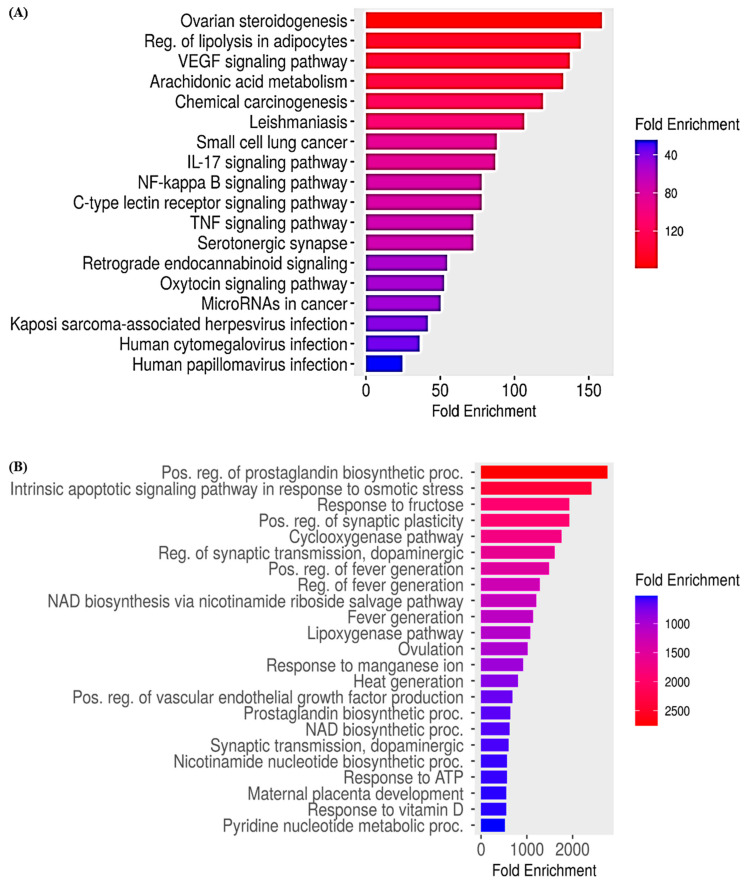
ShinyGO analysis: (**A**) KEGG pathway enrichment, (**B**) Functional enrichment.

In the present study, Figure 8A shows the KEGG pathway enrichment with the fold enrichment scores. In the enrichment analysis, the differentially expressed genes were significantly enriched in ovarian steroidogenesis, regulation of lipolysis in adipocytes, vascular endothelial growth factors (VEGF) signaling pathway, and arachidonic acid metabolism. KEGG pathway analysis indicated that the differentially expressed genes were significantly enriched in metabolic, cancer, inflammatory, and other significant signaling pathways. Similarly, Figure 8B represents the functional enrichment analysis in which the most significantly enriched function was the positive regulation of the prostaglandin biosynthetic process and intrinsic apoptotic signaling pathway in response to osmotic stress. Table 6 and Figure 8 represent the different signaling pathways with fold enrichment scores.

### 3.10. Molecular Simulation Study

**Figure 9:**
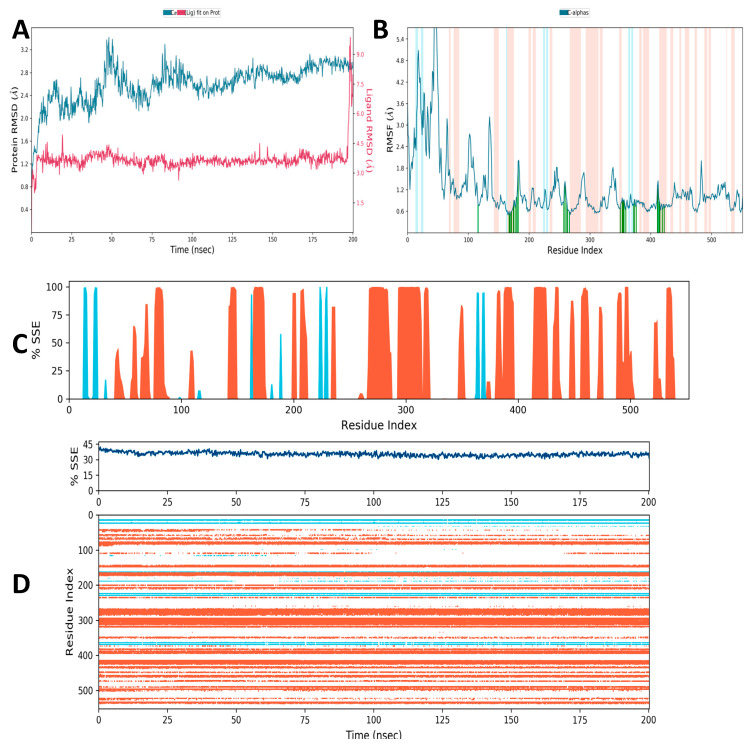
Molecular simulation depicting: (**A**) RMSD, (**B**) RMSF, (**C**,**D**) % SSE of COX-2 protein.

Determining the protein’s RMSD allows us to understand how the protein’s structural conformation has changed over time. This method is particularly well suited for studying minute atomic motions on femto/picosecond timescales, which are extremely challenging to measure experimentally. The RMSD was determined in a simulation of about 200 ns. The average values of RMSD of Cα-COX-2 were determined to be around 2.74 Å in the presence of picrocrocin (Figure 9A). As estimated, the RMSD values and atomic and residual fluctuations of COX-2 were found to be highest in the presence of picrocrocin. The observed structural deviations of COX-2 may be due to the greater number of hydroxyl groups and the presence of a ring structure in picrocrocin. In addition, RMSF values were also plotted against each residue of the COX-2 backbone in the presence of picrocrocin (Figure 9B). As can be seen in Figure 9B, the RMSF plot reveals higher-order residual fluctuations in the presence of picrocrocin. The peaks in the plot represent the protein areas that fluctuate most during the process of simulation. Usually, we have found that the N- and C-terminal tails fluctuate more in comparison to other regions of the protein. This analysis shows that the stability of COX-2 protein has a direct relation with the amino-acid fluctuations.

#### 3.10.1. Secondary Structural Elements

To further investigate this, the effect of picrocrocin on the secondary structural elements (α-helical and β-strand regions) of the COX-2 protein was determined (Figure 9C,D). Helices or strands that last for more than 70% of the simulation as a whole designate these areas. The α-helical and β-strand regions are highlighted with red and blue backgrounds, respectively, and protein SSEs (secondary structure element) are visualized throughout the simulation. The plots in Figure 9C,D depict the residue index distribution throughout the protein structure. Over time, the graphs monitor each residue and SSE assignment, and at the bottom, the plot summarizes the composition of SSE for each trajectory frame during the process of simulation. The strand was 4.22%, while the helix was 31%. The COX-2 simulation times describe the changes in the residue index in the synthesis of protein SSE, such as the blue and red arrows depicting helices and strands. The changes may be due to the breakdown of the hydrogen bond network of COX-2 residues due to the conformational instability of protein in the presence of picrocrocin.

**Figure 10:**
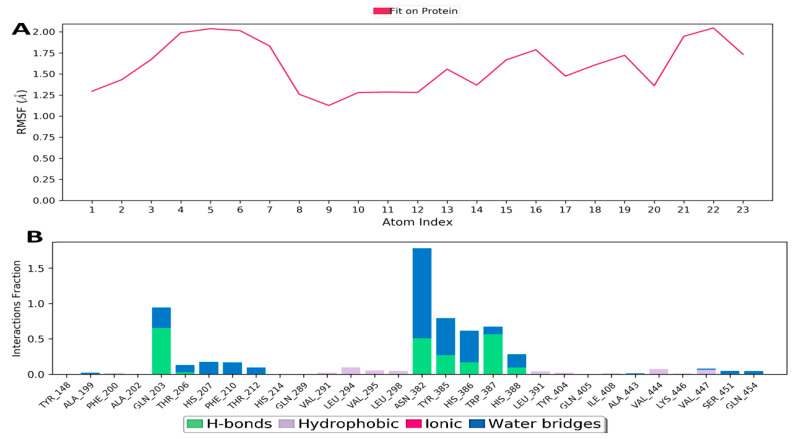
RMSF of picrocrocin ligand: (**A**,**B**) ligand–protein interactions.

Figure 10A shows the determined Ligand Root Mean Square Fluctuation (L-RMSF). This helps in characterizing changes in the ligand atom positions. The mean of the RMSD ligand was found to be 1.5 Å. By breaking down the ligand’s fluctuations into individual atoms, the ligand RMSF displays a 2D structure. In the present study, Figure 10B summarizes the protein-ligand contacts that includes hydrogen bonds (H-bonds), hydrophobic/ionic interactions, water bridges and contacts as a timeline representation.

#### 3.10.2. Hydrogen Bonding

Hydrogen bonding is essential for ligand–protein interaction because it has a significant effect on drug specificity, adsorption, and metabolism, i.e., drug development. The backbone acceptor and donor, side-chain acceptor, and donor are the four types of hydrogen bonding. Various geometric criteria were taken into consideration in hydrogen bonding like distance (2.5 Å) between donor and acceptor atoms, donor angle (^3^120°) between donor and acceptor, and acceptor angle (^3^90°) between acceptor and donor.

#### 3.10.3. Ionic Interaction

Ionic interaction is also known as the polar interaction between protein and ligand and is identified by the presence of metal ions within 3.4 of heavy atoms of ligand and protein (except carbon). Polar interactions are generally of two types, as mediated by the protein backbone and side chain.

#### 3.10.4. Hydrophobic Contacts (like p-p, p-Cation, etc.)

These interactions involve the bonding between hydrophobic amino acid residues of protein with the aliphatic or aromatic carbon groups of ligands. The general criteria for the interactions are p-cation-aromatic and charged groups with 4.5 Å range, p-p—aromatic groups stacked face to face or face to edge, and for other interactions, 3.6 Å range between side chain and carbon groups.

#### 3.10.5. Water Bridge (like Water-Protein and Water-Ligand)

This type of interaction involves a hydrogen bond and a water molecule, but it is less flexible than hydrogen bonding. The criteria involved are a distance of 2.8 Å, ^3^110° donor angle, and ^3^90° acceptor angle.

#### 3.10.6. Protein–Ligand Contacts

**Figure 11:**
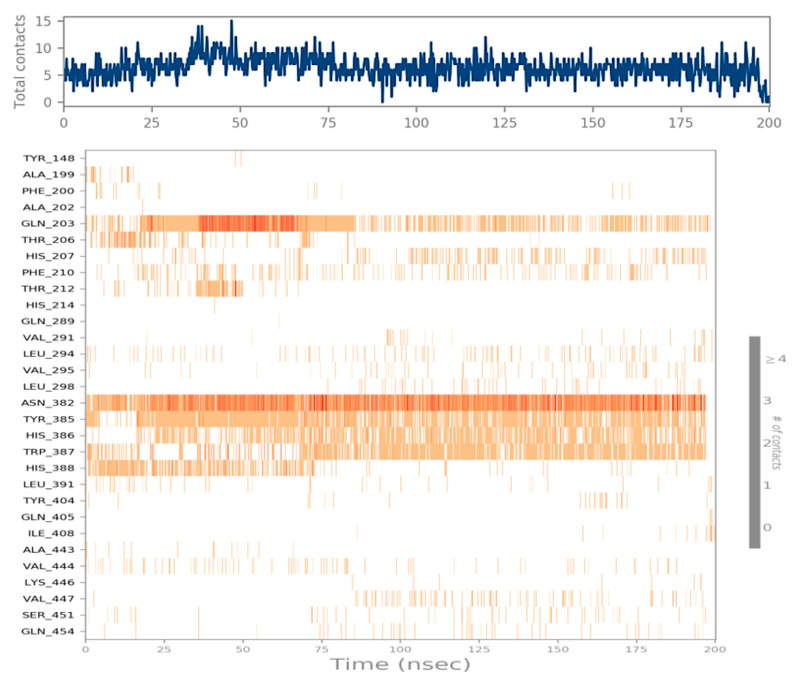
Timeline representation: hydrogen-bonds, water bridges, hydrophobic and ionic interactions.

During this study, the total number of distinct interactions that a protein makes with the ligand is depicted in the top panel of Figure 11. In each trajectory, the lower panel of Figure 11 displays which residue of the protein interacts with the ligand. According to the scale to the right of the plot, the darker orange color indicates that some residues have many specific contacts with the ligand. It can be seen in Figure 11 that more specific interaction was shown by amino acid residues like GLN^203^, TYR^385^, HIS^386 and 388^, ASN^382^, and TRP^387^.

#### 3.10.7. Torsion Profile

**Figure 12:**
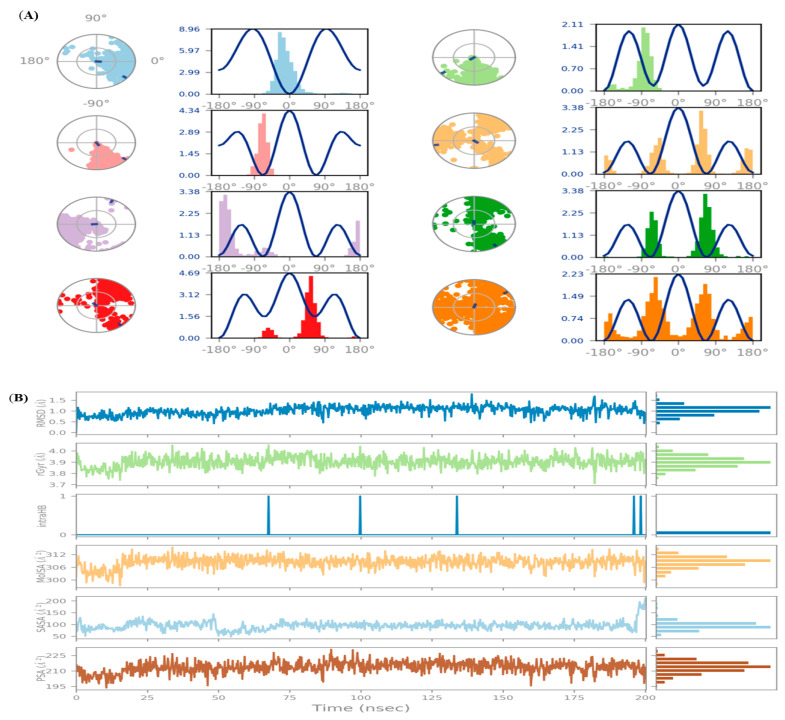
Ligand torsion profile (**A**, Upper panel), and (**B**, Lower panel) ligand properties.

During the simulation trajectory (0 through 200.0 nanoseconds), the conformational evolution of rotatable bonds in the ligand is summarized in the ligand torsion figure. The 2D schematics of a ligand with colored rotatable bonds are displayed in Figure 12A’s top panel, where radial and bar plots (charts) of the same color accompany each rotatable bond torsions. How the torsion has changed is shown by radial (dial) charts, while a bar plot determines the probability density of torsion throughout the simulation. In the center of the radial map, the time evolution is represented radially outwards from the simulation’s start point.

The ligand properties like RMSD, Rg, SASA, PSA, intra-hydrogen bonding, and MoISA are represented in Figure 12B. The charts also show the potential of rotatable bonds with the help of torsional potential. The *Y*-axis of the plot shows the potential values in kcal/mol, and the torsion potential and histogram link might shed light on the ligand conformational strain, which is experienced by the ligand to retain protein-bound conformation. 

## 4. Discussion

COX-2 is an important bi-functional enzyme that mediates prostaglandin synthesis during the inflammatory processes and has become a vital therapeutic target for developing anti-inflammatory medications. Synthetic COX-2 inhibitors hold great potential for treatments; however, drug toxicity creates problems, and as a result, new approaches are required. In such a scenario, natural compounds hold great potential for inhibiting inflammatory signaling pathways including COX-2, which has gained much consideration over the past few years. In this study, natural constituents of saffron (crocin, crocetin, picrocrocin, quercetin, dimethyl crocetin, and rutin) were used. The pertinent association between the identification, synthesis, and purification of compounds during drug discovery has a significant interest [51,52]. In this study, ADMET properties were used to determine the pharmacokinetics of drugs in the body. The five parameters of Lipinski’s rule were used to determine the drug-like properties of compounds, and these features are mostly important as they are related to dissolution and intestinal permeability [39,53,54,55]. In our study, all the molecules followed Lipinski’s rule except rutin, which has a molecular weight >500 g/mol, a number of hydrogen bond donors >5, and a number of acceptors >10. If a compound fails in more than two of Lipinski’s rules and violations, it cannot be consumed and hence used as a drug [39]. The compounds are classified as hydrophilic or hydrophobic when the *logP* scores are negative or positive. A hydrophilic compound has good water solubility, gastric tolerance, and elimination by the kidneys, whereas lipophilic compounds have lower aqueous solubility/gastric tolerance, and good plasma protein binding, elimination, and permeability across the biological membrane [56]. 

During clinical trials, unfavorable effects caused by drug toxicity often lead to drug breakdown [57]. In the process of drug development, it is important to predict the toxicity of compounds, and in such a situation, ProTox-II becomes a particularly significant tool [40]. The estimation of toxicities via computational analysis is much faster than determining toxic doses in animals but also decreases the amount of animal experimentation [58]. The protein targets are the toxicity sites that have been related to adverse drug reactions and effects. Generally, the toxic doses are specified as LD_50_ (mg/kg) of body weight.

Plant compounds with decreased toxicity and good bioavailability have been selected to determine their therapeutic potential against different diseases [59,60]. In structure-based drug designing, scoring functions are particularly important. The ranking of compounds is carried out with the help of docking algorithms as they produce scoring properties and binding poses [61,62]. Insights into protein interactions with phytocompounds are provided via computational docking methods. In the present study, our analysis indicated that the picrocrocin compound of saffron possesses the best binding affinity of −8.1 kcal/mol. Similar findings have reported that the picrocrocin molecule of saffron possesses protection against diseases [63,64]. This can be explained by the fact that the picrocrocin compound of saffron possesses significant pharmacological properties such as antioxidant, anti-inflammatory, antiproliferative, antiangiogenic, and pro-apoptotic properties [65,66,67]. Moreover, picrocrocin is effective against various diseases like cancer, Alzheimer’s, Parkinson’s, memory impairment, epilepsy, and brain ischemia [64,68,69]. The intake of saffron has been found to reduce inflammation by inhibiting the enzymatic activity of cyclooxygenase and improving the number of inflammatory markers (CRP, IL-6, and TNF-α) [66,70]. Saffron contains numerous molecules such as alkaloids, minerals, anthocyanins, carotenoids, glycosides, and flavonoids (rutin, quercetin, and kaempferol) [71].

In the present study, the PROCHECK server was used to evaluate the accuracy and stereochemical properties of the protein structure. This server analyzes the entire protein geometry with residue-by-residue geometry and provides the stereochemical quality of the predicted protein model. In this study, 90.3% of protein residues occupied the favored region, and the structure was acceptable and could be used for further in silico studies [41]. Thus, the stereochemical properties were validated using Ramachandran analysis and the PROCHECK tool, which indicated that the predicted model could be satisfactory.

Protein–protein interaction information is one of the most significant sources for proteome-wide studies [72], especially in network-associated drug design [73] and to understand human disorders [74]. In this scenario, ComPPI-built interactomes provide much wider coverage and offer the construction of highly confident data sets for analysis of protein–protein interaction at the subcellular level. High-confidence interactomes could be developed from optimized interaction and localization scores that could further help in the area of compartment-associated biological processes [75].

In the present study, the CASTp server provides a complete, thorough description of surface regions (pockets and voids) located on the surface of the protein, which are associated with binding events [76]. In a protein structure, the CASTp server, in addition to providing complete information about all atoms involved in their formation, identifies all the voids and pockets. This tool also measures the area and volume of each void and pocket. Furthermore, it measures the size of individual pocket mouth openings, which facilitates determining the availability of binding sites to different ligands and substrates. In several research studies, CASTp computation analysis has proved quite useful [77,78,79].

In this study, motion stiffness is represented by eigenvalues, and its value is proportional to the energy needed to deform the protein structure [80]. The deformation will be easier when the eigenvalues are lower and vice versa. In a protein molecule, the measure of local flexibility is provided through deformation analysis. Atomic displacement (B factors) is dependent on structural heterogeneity and provides a broad range of information about protein structure and dynamics. B-factors enable the study of protein flexibility and biological protein–protein binding locations, as well as the differentiation of crystal packing contacts [81]. The covariance matrix map depicts the related actions of several pairs of anti-correlated and correlated residues, which are represented by white, red, and blue colors that represent residue pair coupling [80].

In research studies, a good method for evaluating binding energies and revealing binding structures is provided by MM-PB(GB)SA [82]. The correctness of docking complex calculations is provided through these rescoring approaches [83,84]. Due to the high efficiency and accuracy of these computational approaches, they have been used mostly in docking calculations. However, a challenging task would be to find more advanced rescoring methods within tolerable limits.

Enrichment analysis shows the set of enriched genes associated with a particular pathway in huge-scale datasets. Functional enrichment analysis provides significant insights into identifying diseases and drug mechanisms. In functional enrichment, gene ontology is the most extensively used. Gene enrichment analysis helps to associate a disease phenotype with a group of genes/proteins, and by identifying vital characteristics, the enrichment analysis can help in the discovery of biological functions. The enrichment analysis predicts whether the fraction of pathway genes of interest is higher in comparison to outside pathway genes.

The molecular dynamic simulation shows that picrocrocin destabilizes the activity of the COX-2 protein by altering its tertiary structure, which causes more pronounced amino-acid fluctuations as determined using RMSD and RMSF values in Figure 9. It was also noted in the simulation studies of the hydrogen bond network that picrocrocin also disrupts the intra-protein hydrogen bond network, thereby causing more unfolding of the COX-2 protein. Furthermore, the direct interaction of picrocrocin with various amino acid residues like GLN^203^, TYR^385^, HIS^386 and 388^, ASN^382^, and TRP^387^ is very crucial for the activity of the COX-2 protein. The modification of these residues with overall structural alterations in protein presents the potential inhibitory properties of this compound; thus, picrocrocin might prove to be a potential therapeutic moiety for the treatment of various disorders like inflammation.

## 5. Conclusions

In silico approaches were applied to the compounds of saffron to reveal their potential against the human COX-2 protein. Our molecular docking study suggested that picrocrocin of saffron was the most potent inhibitor of the COX-2 protein. In our study, the ADMET analysis revealed the physiochemical properties and the toxicity of the compounds of saffron, while the quality and correctness of the COX-2 protein structure were predicted using molecular modeling, which predicted 90.3% of COX-2 residues as lying in the Ramachandran favored region. We know that protein–protein interactions play a significant role in the cellular functions of the living organism, and thus the protein–ligand interaction and behavior of protein motion can be determined using molecular dynamics approaches. The molecular simulation studies showed that there are overall conformational alterations with a decrease in the hydrogen bond network in the COX-2 protein in the presence of picrocrocin. Overall, compared to the currently available methods, the picrocrocin used in this study could serve as an alternative for use against inflammatory diseases. However, more research studies are required to validate the inflammatory role of picrocrocin. Thus, based on these findings, we can conclude that picrocrocin can be repurposed as a drug candidate against inflammatory diseases.

## Figures and Tables

**Table 1:** ADMET analysis of saffron constituents.

ADMET	Physiochemical Properties					
	Compounds				Drugs	
	Crocetin	Picrocrocin	Quecertin	Rutin	Aspirin	Rofecoxib
Molecular Weight (g/mol)	328.40	330.37	302.24	610.52	180.16	314.36
Topological polar surface area (TPSA) Å^2^	74.60	116.45	131.36	269.63	63.60	68.82
Hydrogen bond acceptors	4	7	7	16	4	4
Hydrogen bond donors	2	4	5	10	1	0
Molar refractivity	98.48	81.08	78.03	141.8	44.90	83.69
XLOGP	5.41	−0.50	1.54	−0.33	1.19	2.27
iLOGP	3.33	2.07	1.63	0.46	1.30	2.13
MLOGP	3.52	−0.88	−0.56	−3.89	1.51	2.62
WLOGP	4.61	−0.49	1.99	−1.69	1.31	3.64
Lipinski	Yes	Yes	Yes	No	Yes	Yes
GI absorption	High	High	High	Low	High	High
Bioavailability score	0.85	0.55	0.55	0.17	0.85	0.55
BBB permeability	No	No	No	No	Yes	Yes
Pgp substrate	No	Yes	No	Yes	No	No
CYP2D6 inhibitor	No	No	Yes	No	No	No
PAINS (Pan assay interference compounds)	0	0	1	1	0	0
Leadlikeness	No	Yes	Yes	No	No	Yes
Skin permeation (log kp) cm/s	−4.46	−8.67	−7.05	−10.26	−6.55	−6.61

**Table 2:** Predicted toxicity analysis of compounds and drugs.

Compounds	Toxicity Class	Hepatotoxicity	Immunotoxicity	Carcinogenicity	Mutagenicity	Cytotoxicity
Crocetin	5	0.72	0.99	0.74	0.72	0.72
Picrocrocin	3	0.83	0.83	0.78	0.73	0.87
Quercetin	3	0.69	0.87	0.68	0.51	0.99
Rutin	5	0.80	0.98	0.91	0.88	0.64
Aspirin	3	0.51	0.99	0.86	0.97	0.94
Rofecoxib	5	0.55	0.97	0.71	0.68	0.76

**Table 3:** Selected saffron compounds with their Ki and binding affinity.

S. No.	Phytocompounds	Binding Affinity (kcal/mol)	Ki (µM)
1	Crocetin	−7.4	12.50
2	Picrocrocin	−8.1	13.68
3	Quecertin	−7.6	12.87
4	Rutin	−7.9	13.38
5	Safranal	−6.8	11.51
6	Crocin	−7.1	12.02
7	Dimethylcrocetin	−7.2	12.19
	**Drug**		
1	Aspirin	−6.1	10.30
2	Rofecoxib	−8.3	14.05

**Table 4:** Position and interactions between chosen protein and compounds.

Compound	Type of Interaction	Position of Interaction
Crocetin	Hydrogen bonding	ASN34, ARG44
	Van der Waals	PRO154, PRO156, TYR136, TYR130, GLY135, GLY45, GLN461, VAL46, CYS36, CYS47, ARG469, ASP125
	Alkyl/Pi-alkyl	PRO153, HIS39, LEU152
Picocroxin	Hydrogen bonding	TYR373, GLN374, SN375
	Van der Waals	GLY225, HIS226, ARG376, LEU145, ASN375, PHE142, PRO127, TYR373
Quercetin	Hydrogen bonding	CYS47, TYR130, ARG469, ARG44, ASP125
	Van der Waals	PRO40, HIS39, GLN461, LEU152, GLY45, GLY135, CYS41, CYS36, MET48
	Pi-alkyl	PRO153
Rutin	Hydrogen bonding	SER126, HIS122, PHE371, LEU366, TYR373, THR118
	Van der Waals	LYS369, THR62, THR60, LYS560, GLN370, SER121, PHE367, ARG44, GLN372, LYS369, LYS532, ILE124, ASP125, ALA543, LEU123
	Alkyl/Pi-alkyl	ARG61, PRO542
**Drug**		
Aspirin	Hydrogen bonding	TRP139, ASP229, ARG333
	Van der Waals	LEU224, GLY235, GLU236, THR237, LEU238, GLN241
Rofecoxib	Hydrogen bonding	ARG44
	Van der Waals	SER121, ALA543, LYS532, ASP125, SER126, PHE371, HIS122, GLN370, GLN372, TYR373
	Pi-alkyl	PRO542

**Table 5:** MM/PB(GB)SA free binding energies of human COX-2 protein.

ELE	VDW	GAS	PBSOL	PBTOT	GBSOL	GBTOT	TS	Delta PB	Delta GB
−271.82	−74.73	−346.55	351.66	5.12	352.92	−20.63	37.45	42.57	16.82

**Table 6:** Pathway analysis with fold enrichment scores.

FDR Enrichment	Genes of Pathway	Gene	Fold Enrichment	Name of Pathway
0.025356055	51	1	158.7843137	Ovarian steroidogenesis
0.025356055	56	1	144.6071429	Reg. of lipolysis in adipocytes
0.025356055	59	1	137.2542373	VEGF signaling pathway
0.025356055	61	1	132.7540984	Arachidonic acid metabolism
0.025356055	68	1	119.0882353	Chemical carcinogenesis
0.025356055	76	1	106.5526316	Leishmaniasis
0.025356055	92	1	88.02173913	Small cell lung cancer
0.025356055	93	1	87.07526882	IL-17 signaling pathway
0.025356055	104	1	77.86538462	NF-kappa B signaling pathway
0.025356055	104	1	77.86538462	C-type lectin receptor signaling pathway
0.025356055	112	1	72.30357143	TNF signaling pathway
0.025356055	112	1	72.30357143	Serotonergic synapse
0.029159463	148	1	54.71621622	Retrograde endocannabinoid signaling
0.029159463	154	1	52.58441558	Oxytocin signaling pathway
0.029159463	161	1	50.29813665	MicroRNAs in cancer
0.032940232	194	1	41.74226804	Kaposi sarcoma-associated herpesvirus infection
0.035796784	224	1	36.15178571	Human cytomegalovirus infection
0.049957465	331	1	24.4652568	Human papillomavirus infection

## Data Availability

All the data generated has been published in this manuscript.
